# Inhibition of *β-*Glucocerebrosidase Activity Preserves Motor Unit Integrity in a Mouse Model of Amyotrophic Lateral Sclerosis

**DOI:** 10.1038/s41598-017-05313-0

**Published:** 2017-07-12

**Authors:** Alexandre Henriques, Mylene Huebecker, Hélène Blasco, Céline Keime, Christian R. Andres, Philippe Corcia, David A. Priestman, Frances M. Platt, Michael Spedding, Jean-Philippe Loeffler

**Affiliations:** 1 Université de Strasbourg, UMR_S 1118, Fédération de Médecine Translationnelle, Strasbourg, France; 2 INSERM, U1118, Mécanismes Centraux et Périphériques de la Neurodégénérescence, Strasbourg, France; 3 Spedding Research Solutions SAS, Le Vesinet, France; 4 Department of Pharmacology, University of Oxford, Oxford, UK; 5 INSERM, Université François-Rabelais, U930, Neurogénétique et Neurométabolomique, Tours, France; 6 CHRU de Tours, Laboratoire de Biochimie et de Biologie Moléculaire, Tours, France; 7 IGBMC (Institut de Génétique et de Biologie Moléculaire et Cellulaire), INSERM, U964, CNRS, UMR7104, Université de Strasbourg, 67404 Illkirch, France; 8 CHRU de Tours, Centre SLA, Tours, France

## Abstract

Recent metabolomic reports connect dysregulation of glycosphingolipids, particularly ceramide and glucosylceramide, to neurodegeneration and to motor unit dismantling in amyotrophic lateral sclerosis at late disease stage. We report here altered levels of gangliosides in the cerebrospinal fluid of amyotrophic lateral sclerosis patients in early disease stage. Conduritol B epoxide is an inhibitor of acid beta-glucosidase, and lowers glucosylceramide degradation. Glucosylceramide is the precursor for all of the more complex glycosphingolipids. In SOD1^G86R^ mice, an animal model of amyotrophic lateral sclerosis, conduritol B epoxide preserved ganglioside distribution at the neuromuscular junction, delayed disease onset, improved motor function and preserved motor neurons as well as neuromuscular junctions from degeneration. Conduritol B epoxide mitigated gene dysregulation in the spinal cord and restored the expression of genes involved in signal transduction and axonal elongation. Inhibition of acid beta-glucosidase promoted faster axonal elongation in an *in vitro* model of neuromuscular junctions and hastened recovery after peripheral nerve injury in wild type mice. Here, we provide evidence that glycosphingolipids play an important role in muscle innervation, which degenerates in amyotrophic lateral sclerosis from the early disease stage. This is a first proof of concept study showing that modulating the catabolism of glucosylceramide may be a therapeutic target for this devastating disease.

## Introduction

Amyotrophic lateral sclerosis (ALS) is a severe neurodegenerative disease and the most common motor neuron disease among adults. ALS is characterized by a loss of upper motor neurons in the motor cortex and of lower motor neurons located in the brainstem and in the spinal cord. Gene mutations are associated with familial and sporadic forms of ALS. Most of the genetic forms of ALS are associated to mutations in genes encoding superoxide dismutase 1 (SOD1), TAR DNA binding protein of 43-kDa (TDP-43) and fused in sarcoma (FUS), or to hexanucleotide repeat expansions in chromosome 9 open reading frame 72 (C9ORF72)^1^. The therapeutic options for ALS remain limited despite extensive preclinical and clinical research leading to more than 50 randomized clinical trials, a wide majority of them aiming to counteract directly the process of neurodegeneration^2^. Along with the loss of motor neurons, a metabolic pathology is present in ALS and affects the central nervous system and peripheral organs^3^. A high incidence of dyslipidemia and hypermetabolism is present in ALS patients^4, 5^. High LDL/HDL ratios or high body mass index are associated with better prognosis and slower disease progression^6, 7^. Pilot clinical trials suggest that nutritional diets enriched with lipids are beneficial for ALS patients in terms of survival and for preventing weight-loss^8–10^. The SOD1 mouse, a genetic model of ALS, is hypermetabolic, and uses lipids preferentially over carbohydrates as nutrients^11^, and survives longer when fed with a high fat diet^12^. ALS is thus a disease with a major metabolic component.

Changes in levels of sphingolipids, particularly of glycosphingolipids, were recently described in the central nervous system of ALS patients at disease endpoint and in SOD1 mice at various disease stages^13, 14^. With the exception of asialo-GM1 and asiolo-GM2, gangliosides are a subset of glycosphingolipids containing three or more sugar moieties and one or more sialic acids. Conversion of ceramide into glucosylceramide (GlcCer) is the initial step for the *de novo* synthesis of all of the more complex glycosphingolipids, which takes place in the Golgi apparatus^15^. Gangliosides are present in tissues and body fluids, and are particularly enriched in the cell membrane within the nervous system^16, 17^. The composition of brain gangliosides changes from simple (e.g. GM3) to complex gangliosides (e.g. GM1a) during neuronal development, and is associated with increased neurogenesis, synaptogenesis and axonal arborisation^18, 19^.

GM1a is one of the main gangliosides in the nervous system. The mature nervous system requires gangliosides, including GM1a, in lipid rafts and at synapses for maintaining physiological functions. GM1a is anchored in the outer leaflet of the membrane by ceramide linked to five specific sugars which are exposed at the extracellular surface, and can interact with the receptors of growth factors, such as BDNF^20, 21^. Guillain-Barré syndrome is caused by the presence of autoimmune antibodies which target complex gangliosides. Autoantibodies binding to GM1a or to GD1a at nodes of Ranvier and at the presynaptic part of neuromuscular junctions (NMJs) lead to acute motor axonal neuropathy, a sub-type of Guillain-Barré syndrome with severe motor axonal degeneration^22, 23^. GM1a is also located in the nuclear envelope, where it interacts with the sodium-calcium exchanger to potentiate calcium transfer and can regulate genes during neuronal development by binding to acetylated histones^24, 25^.

We have shown recently that NMJ integrity in SOD1^G86R^ mice requires functional synthesis of glucosylceramide, the precursor of more complex glycosphingolipids, suggesting that they could be key modulators of ALS severity^13, 14^. Indeed, inhibition of GlcCer synthesis caused impaired nerve regeneration after peripheral nerve injury and was highly deleterious in SOD1^G86R^ mice^13, 14^. The expression of *ugcg*, a gene encoding the enzyme catalysing the first step of glycosphingolipid synthesis, was strongly upregulated in muscle biopsies of ALS patients, independent of disease severity, suggesting a dysregulation in glycosphingolipid homeostasis in ALS at early disease stage.

Since inhibition of the synthesis of GlcCer delayed motor recovery in wild type mice and caused fragmentation of motor endplates in SOD1^G86R^ mice, we reasoned that inhibition of β-glucocerebrosidase (GCase), which is responsible for the hydrolysis of GlcCer, may be beneficial in SOD1^G86R^ mice. We thus used a well-characterised specific and covalent GCase inhibitor, conduritol B epoxide (CBE)^26^. High dose CBE (100 mg/kg/d) has been used to inhibit the lysosomal GCase almost completely in neonatal mice to induce a chemical model of Gaucher’s diseases with neuronal toxicity^27, 28^. However, low dose CBE (10 mg/kg/d) triggers partial inhibition, allowing residual activity and rapid wash-out with no reported toxicity in adult mice^26, 28^. We studied the metabolism of glycosphingolipids in ALS at early disease stage, in patients and in SOD1^G86R^ mice, and its relation to the integrity of NMJs.

## Results

### Altered ganglioside expression is evident at disease onset in SOD1^G86R^ mice and ALS patients

Gangliosides, particularly GM1a, are key structural components of the myelin sheath and also of lipid rafts at the cell surface. We sought to determine whether disease progression could be associated with loss of GM1a at the neuromuscular junctions (NMJs) in an animal model of ALS, the SOD1^G86R^ mouse. The symptomatic phase of the disease starts at 95 days of age in this animal model of ALS. Symptoms include muscle weakness, mild locomotor impairments and muscle fibrillation detectable by electromyography. Symptoms progress towards skeletal muscle paralysis and atrophy, leading to death around 105 days of age.

Distribution of GM1a and other gangliosides was studied at the NMJs of SOD1^G86R^ mice, using the cholera toxin subunit B (CTB), which is known to bind to gangliosides with highest affinity for GM1a^29, 30^.

**Figure 1:**
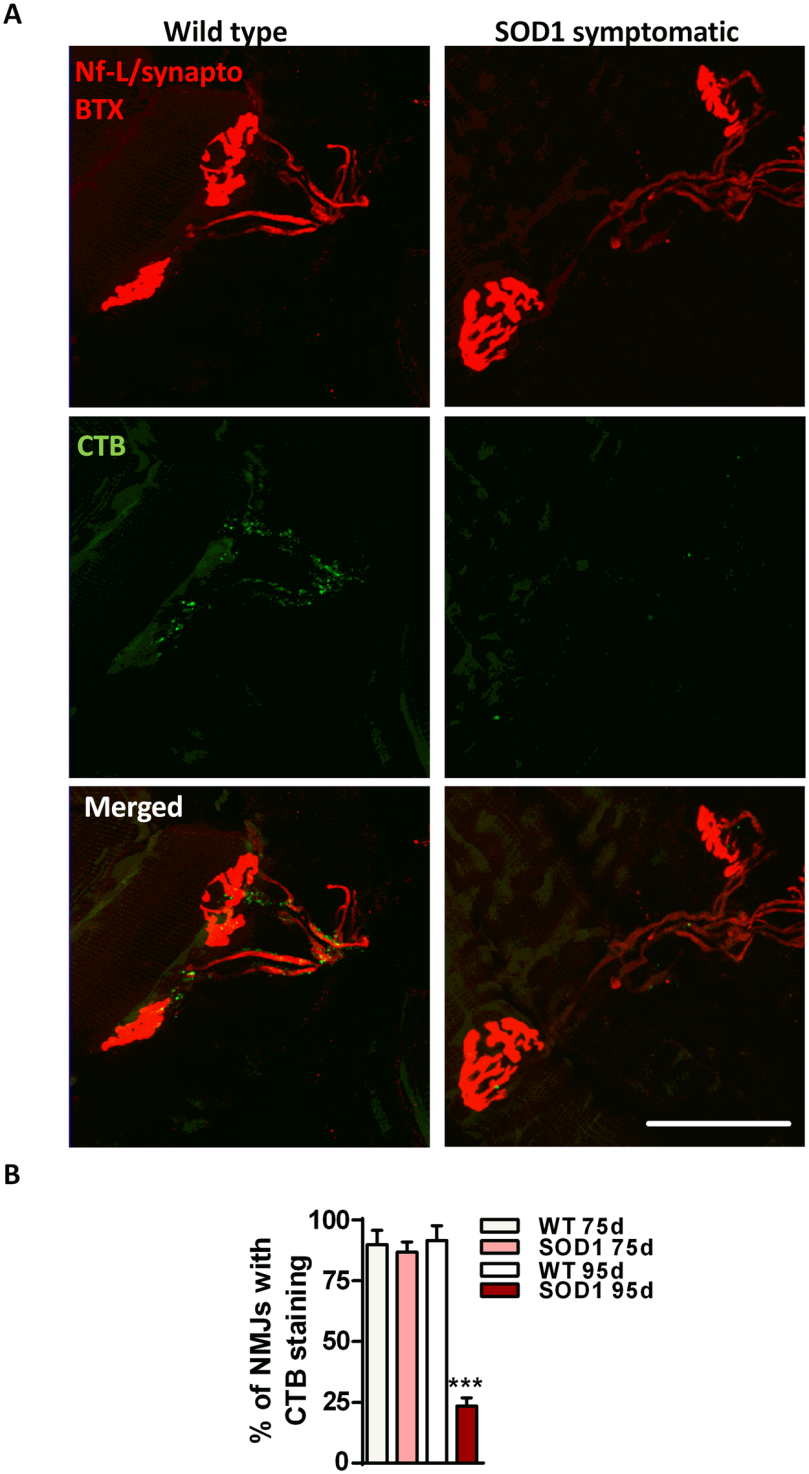
Loss of CTB staining at the NMJs in the tibialis anterior muscle of early symptomatic SOD*1* ^*G86R*^ mice. (**A**) Representative pictures of ganglioside distribution along motor axons, after staining with cholera toxin subunit B (CTB, green) and with neurofilament-L, synaptophysin and bungarotoxin (red). Scale bar = 50 µm. (**B**) Quantification showing the proportion of NMJs with normal cholera toxin subunit B (CTB) staining along motor axons in presymptomatic and early symptomatic SOD1^G86R^ mice (**B**). WT 75d n = 5, SOD1 75d n = 5, WT 95d n = 5, SOD1 95d n = 6; ***p < 0.001. Mean ± SEM.

CTB signal was detected close to the motor endplates in wild type controls (Fig. 1A). Most of the structures were localized in close proximity to the axonal proteins neurofilament-L and synaptophysin, which are found in motor axons innervating NMJs. CTB staining was detected close to the post-synaptic part of NMJs, where CTB-positive structures overlapped with clusters of acetylcholine receptors (Fig. 1A). Distribution of CTB-positive structures was further studied at the presynaptic and post-synaptic parts of NMJs in SOD1^G86R^ mice. At 75 days of age, before disease onset, CTB staining was evident close to all NMJs and had a distribution pattern comparable to wild type mice. At 95 days of age, at disease onset, when the very first motor symptoms were detected, the majority of correctly innervated NMJs showed reduced axonal CTB staining in SOD1^G86R^ mice (Fig. 1B and Supp. Fig. 1), indicating that ganglioside dysregulation is coincident with the loss of the NMJs, and may start before widespread denervation.

Cerebrospinal fluid plays a major role in the clearance of neuronal and axonal debri^31^. Individuals suffering from demyelinating diseases or from severe neuronal loss have increased sphingolipid content (e.g. sphingomyelin, ceramide and glycosphingolipids) in their cerebrospinal fluid^32, 33^. In an attempt to detect neuronal or axonal loss of gangliosides, we sought to quantify their levels in the cerebrospinal fluid of ALS patients at diagnosis and of individuals diagnosed with non-demyelinating neurological disorders. Although not significant, a trend for increased protein level was detected in the samples from ALS presented as compared to controls, which is often seen in neurodegenerative diseases^34^. GlcCer, and gangliosides from the a-series (GM1a and GD1a) and b-series (GD1b and GT1b) were identified by their sugar moieties using high performance liquid chromatography **(**Table 1 and Supp. Fig. 2). The levels of other gangliosides were below the threshold for quantification. Levels of GlcCer and GM1a were both significantly increased in ALS patients. None of the levels of the glycosphingolipids correlated with the age at onset or BMI (data not shown). The severity of progression of the disease, determined by the loss of ALSFRS-R score per month, was available for 11 ALS patients. In this subset of patients, the severity of the disease was positively correlated with the level of GM1a in the cerebrospinal fluid (Supp. Fig. 2), maybe indicating neuronal loss or elevated basal levels of GM1a, which could be detrimental for the prognosis of patients. Taken together, these results show that the homeostasis of gangliosides is dysregulated early on in ALS.

**Table 1:** Glycosphingolipid dysregulation in the cerebrospinal fluid of ALS patients.

Items	Neurological disorders	ALS	*p*-value
***Cohort size***	12	14	
*Female* (%)	33.3	40	
*Male* (%)	66.7	60	
***Age (years)***	68.3 ± 1.4	70.3 ± 0.6	n.s.
***ALSFRS-R***	N/A	39.9 ± 0.3	—
***Site of onset*** **(** ***bulbar***/***spinal*** **)**	N/A	5/9	—
***CSF glucose*** **(** ***mmol***/***L*** **)**	3.8 ± 0	4.1 ± 0	n.s.
***CSF protein*** **(** ***g*** **/** ***L*** **)**	0.44 ± 0.01	0.51 ± 0.02	n.s.
***GlcCer***	84.8 ± 1	98.9 ± 1.1	*
***GM1a***	83.9 ± 1.1	104.3 ± 2.3	*
***GD1a***	74.6 ± 1.8	88.2 ± 2.3	n.s.
***GD1b***	71.7 ± 1.7	97 ± 2.3	n.s.
***GT1b***	54 ± 2.3	66.8 ± 3.7	n.s

The characteristics and levels of glycosphingolipids of individuals diagnosed with non-ALS neurological disorders and ALS are shown. Glycosphingolipids are expressed as HPLC peak area/µl CSF, Mean ± SEM. *Corrected *p*-value < 0.05.

### Inhibition of GCase activity delays disease onset and improves motor functions in early symptomatic SOD1^G86R^ mice

**Figure 2:**
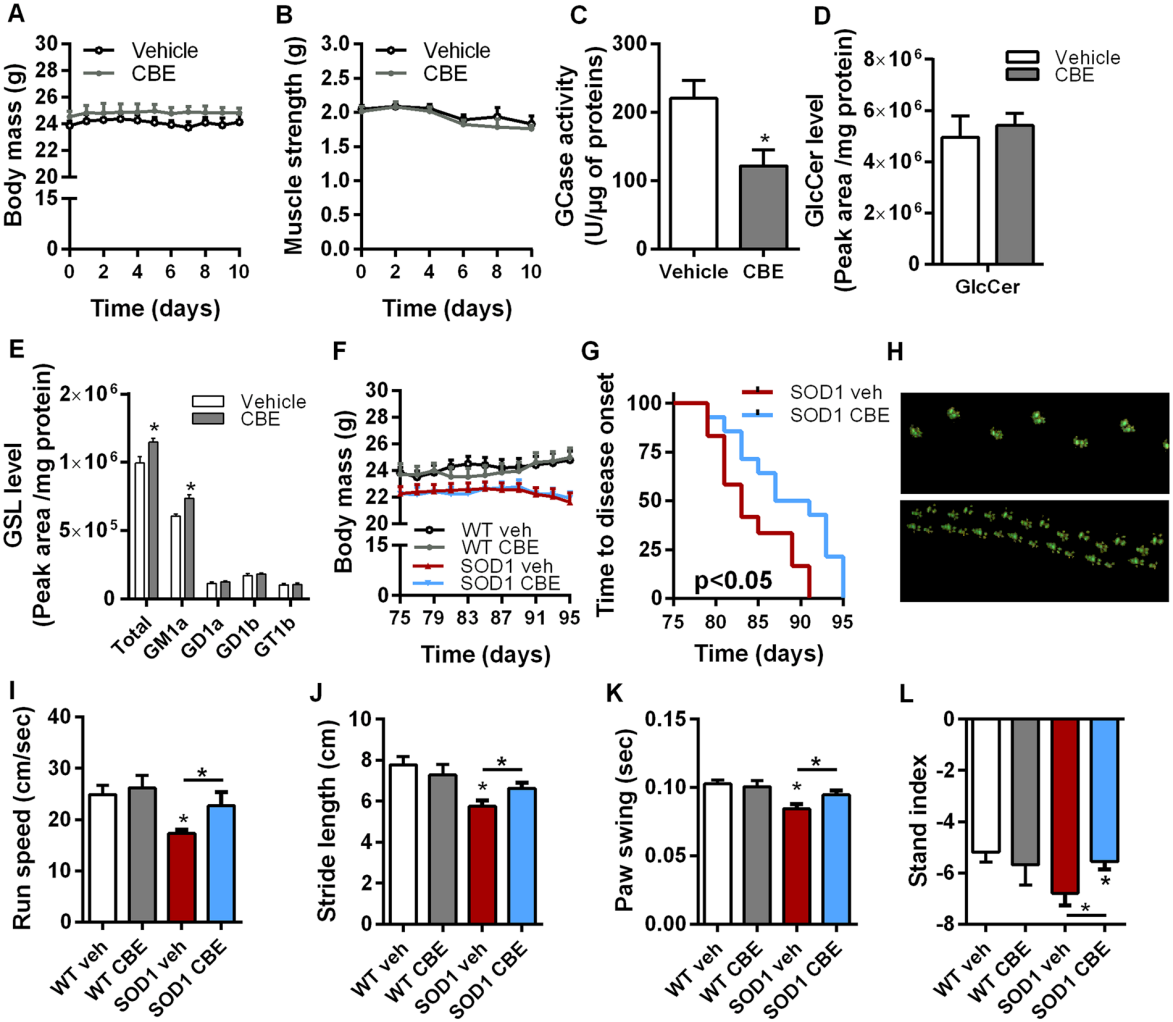
Pharmacological inhibition of GCase activity delays disease onset and improves motor functions in SOD1^G86R^ mice. (**A**) Body mass evolution in wild type mice after 10 days of CBE treatment (n = 5/group). (**B**) Muscle strength of wild type mice after 10 days of CBE treatment (n = 5/group). (**C**) Total β-glucocerebrosidase activity measured in the spinal cord lysate of wild type mice (n = 5). (**D**,**E**) GlcCer (**D**) and gangliosides (**E**) levels in the spinal cord of wild type mice after CBE treatment (n = 5/group). (**F**) Body mass evolution in SOD1 and WT mice (n = 7 for WT groups, n = 12 for SOD1 veh and n = 14 for SOD1 CBE). (**G**) Kaplan-Meier showing time to onset of muscle strength loss in SOD1^G86R^ mice (n = 12 for SOD1 veh and n = 14 for SOD1 CBE, p < 0.02). (**H**–**L**) Catwalk analysis showing representative pattern of strides of symptomatic SOD1^G86R^ mice (**H**), and average speed (**I**), stride length (**J**), paw swing (**K**) and stand index (**L**) in 95 days old mice. Mean ± SEM, *p < 0.05.

Inhibition of GlcCer synthesis weakens NMJs and hastens disease progression in SOD1^G86R^ mice^13, 14^. Here, we sought to prevent the hydrolysis of GlcCer by inhibiting β-glucocerebrosidase activity (GCase). We used conduritol B epoxide (CBE)^26^, known to bind to both the lysosomal and non-lysosomal forms of GCase and to inhibit their activity^35^. Initially, we showed that the CBE treatment (10 mg/kg/day) did not alter body mass and muscle strength of wild type mice (Fig. 2A,B). The treatment successfully inhibited GCase activity and increased total ganglioside levels, mainly through increased ganglioside GM1a in the spinal cord (Fig. 2A–E). Presymptomatic SOD1^G86R^ mice and wild type littermates were then treated with CBE for 20 days. The treatment was terminated at the early disease stage, at 95 days of age, when SOD1^G86R^ mice usually present muscle weakness and denervation.

Body mass, muscle strength and locomotion profiles were monitored to determine the onset of motor symptoms. No change in body weight was observed over the course of the disease after treatment (Fig. 2F). However, the onset of muscle strength loss was significantly delayed by 6 days in SOD1^G86R^ mice treated with CBE compared to the vehicle group (Fig. 2G). Automated gait analysis revealed that the locomotor profile of SOD1^G86R^ mice at 95 days of age was characterized by reduced average speed, reduced stride length and paw swing, compared to wild type littermates. CBE treatment prevented the development of locomotor impairments in SOD1^G86R^ mice and improved speed and step cycles (Fig. 2H–L).

**Figure 3:**
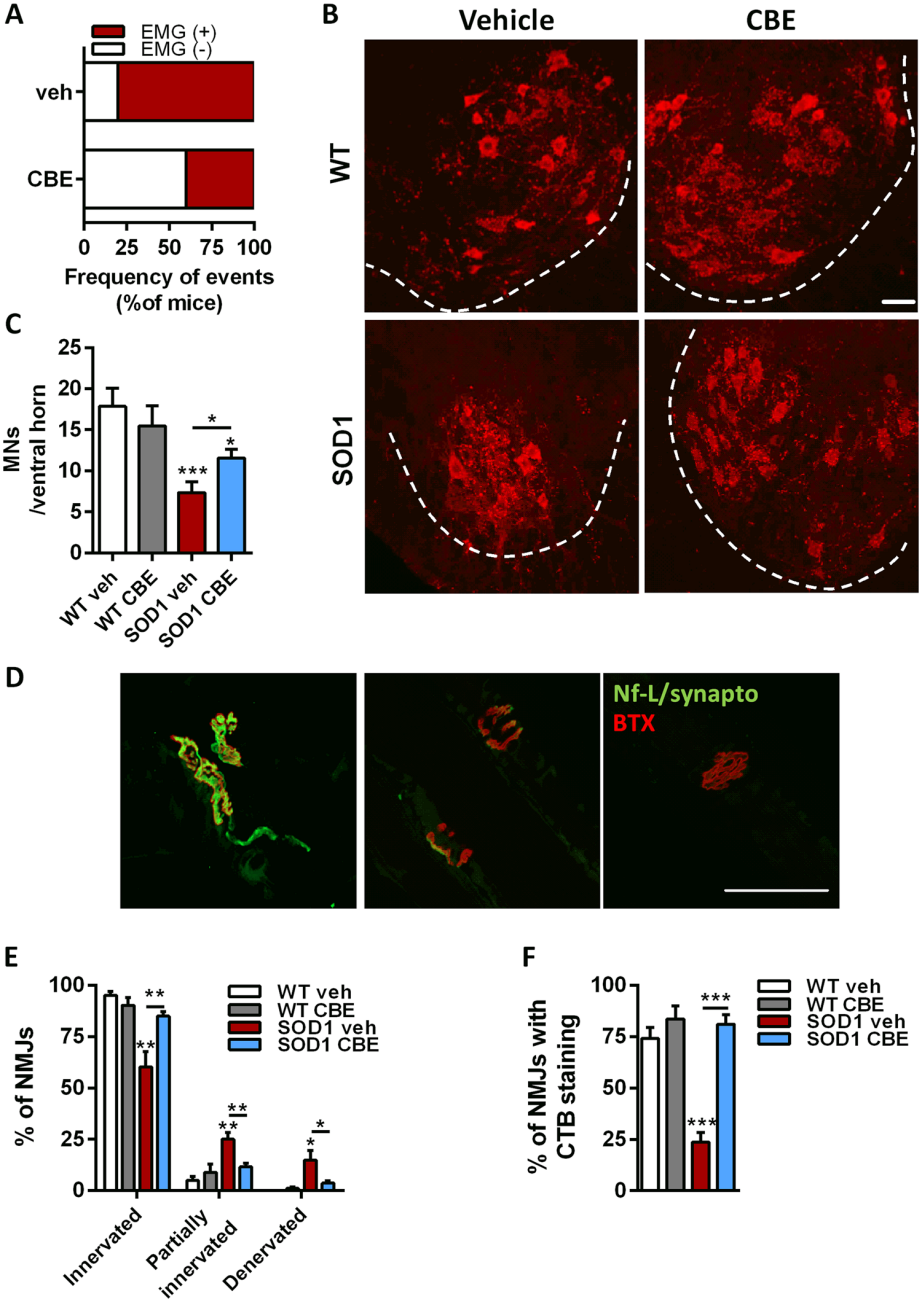
Pharmacological inhibition of GCase activity promotes neuroprotection in SOD1^G86R^ mice. (**A**) Frequency of denervation in gastrocnemius muscle of SOD1^G86R^ mice, at 95 days of age, detected by electromyography (n = 12, SOD1 veh; n = 13 SOD1 CBE). (**B**) Representative pictures of spinal motor neurons, after immunostaining with choline acetyl transferase (ChAT, red). Scale bar = 50 µm. (**C**) Quantification of cells located in the ventral horn of the spinal cord and having a size bigger than 400 µm^2^ (n = 10/group). (**D**) Representative pictures of fully innervated (left), partially innervated (middle) and denervated (right) neuromuscular junctions, after immunostaining with neurofilament and synaptophysin antibodies (green) and bungarotoxin (red), in tibialis anterior muscle. (**E**) Neuromuscular junction integrity in tibialis anterior muscle (n = 10/group). (**F**) Distribution of CTB signal at neuromuscular junctions in tibialis anterior muscle (n = 10/group). Mean ± SEM, *p < 0.05; **p < 0.005; ***p < 0.001.

At 95 days of age, electromyography revealed that muscle fibrillation, a sign of muscle denervation, was less frequently detected in the gastrocnemius muscle of SOD1^G86R^ mice upon CBE treatment, as compared to the vehicle group (Fig. 3A). At this time point, neurodegeneration was detected in the lumbar spinal cord of SOD1^G86R^ mice, and resulted in a 65% loss of large motor neurons as compared to wild type (Fig. 3B,C) and clear signs of denervation were present in tibialis muscle as only 60% of NMJs were fully innervated (Fig. 3D,E). Although still observed, loss of alpha-motor neurons was mitigated by CBE treatment of SOD1^G86R^ mice. Moreover, the number of correctly innervated NMJs was significantly higher in SOD1^G86R^ mice after CBE treatment as compared to the vehicle group **(**Fig. 3E), suggesting that CBE given before disease onset successfully delayed muscle denervation in SOD1^G86R^ mice. Moreover, the treatment prevented loss of CTB-positive structures, potentially lipid rafts containing GM1a and other gangliosides, at NMJs (Fig. 3F). Taken together, these data demonstrate that inhibition of GCase activity by CBE is able to modulate the pathophysiology of ALS and improves the integrity of motor units in SOD1^G86R^ mice.

### Inhibition of GCase activity modulates spinal transcriptome in early symptomatic SOD1^G86R^ mice

**Figure 4:**
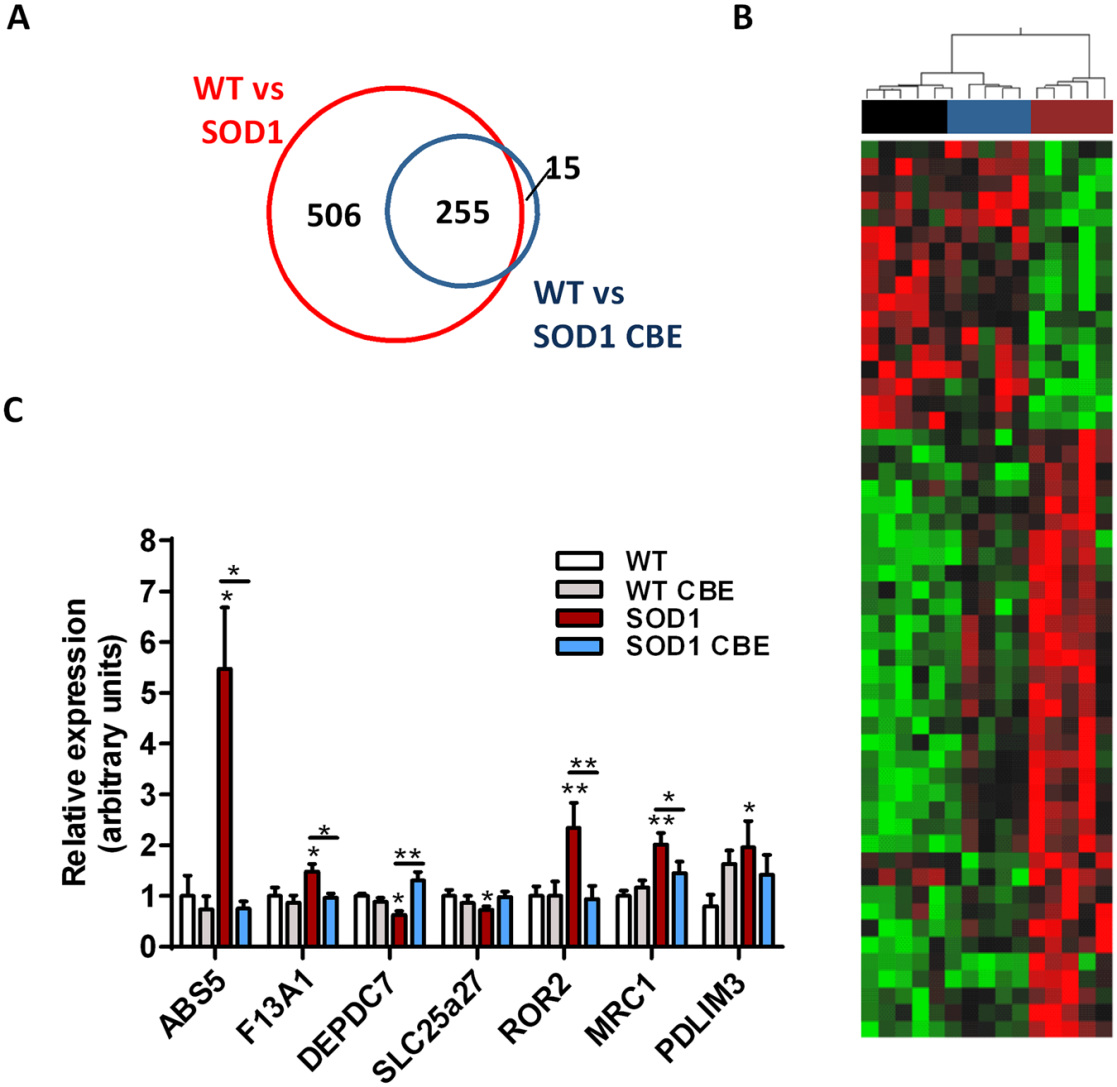
CBE treatment corrects transcriptomic dysregulation in spinal cord *of SOD1* ^*G86R*^ mice. (**A**) Venn diagrams showing gene dysregulations in the spinal cord in SOD1^G86R^ mice receiving the vehicle (red circles) or the CBE (blue circles), and compared to wild type. (**B**) Hierarchical clustering performed with genes significantly regulated by CBE in SOD1^G86R^ mice in the spinal cord (black: wild type; blue: SOD1^G86R^ -CBE; dark red: SOD1^G86R^ veh). (**C**) Expression levels determined by quantitative PCR for selected genes. Data are presented as Mean +/− SEM; *p-value < 0.05.

At 95 days of age, sequencing of spinal messenger RNA identified a total of 761 significantly dysregulated genes in early symptomatic SOD1^G86R^ mice when compared to wild type. The number of abnormalities found in SOD1^G86R^ mice was strongly reduced after CBE treatment (Fig. 4A).

Comparison of transcriptomes from the SOD1 vehicle and SOD1 CBE groups revealed that the treatment had a significant effect on 51 genes (17 upregulated and 36 dowregulated). Most of these genes had readjusted expressions toward wild type levels (Fig. 4B). Over-representation analysis showed that CBE had an effect on the expression of genes associated with “cell cycle”, “hemostasis”, “RET signalling”, “IGF1R signalling cascade” and “axon guidance” (Supp. Table 1). Validation of RNA sequencing was performed by quantitative PCR for a subset of genes (Fig. 4C). Selected genes were (i) found to be dysregulated in SOD1^G86R^ mice compared to wild type, (ii) modulated by CBE and (iii) were associated with relevant pathophysiological features of ALS, such as altered regulation of energy metabolism, inflammation, regulation of ubiquitin-protease system and excitotoxicity/glutamatergic signalling.

The ASB5 gene was strongly dysregulated in the SOD1^G86R^ mice. The function of ASB5 is not well characterized, particularly in the central nervous system. This protein interacts with proteins involved in the maturation of ribosomes, the ribophorins, located in the endoplasmic reticulum, and with E3 ubiquitin ligase^36^. CBE treatment completely restored the expression of ASB5 to basal levels.

MRC1 and F13A1 are two genes coding for proteins that are markers for the M2 phenotype of microglia/macrophage lineage^37, 38^. Upregulation of microglial/macrophage markers in SOD1^G86R^ mice could be a sign of infiltration of peripheral macrophages or activation of microglia cells in symptomatic SOD1^G86R^ mice. CBE treatment had a significant correcting effect on the expression of both genes.

Slc25a27 is the gene coding for uncoupling protein 4, a mitochondrial protein associated with the regulation of mitochondrial membrane potential. UCP4 activity contributes to the uptake of glucose in neurons and the reduction of reactive oxygen species. UCP4 activity is also associated with increased resistance to neuronal stress^39^. In SOD1^G86R^ mice, UCP4 was downregulated, presumably because of altered energy metabolism in ALS^3^, and corrected upon CBE treatment.

ROR2, which was upregulated in SOD1^G86R^ mice, is associated with regulation of synaptic plasticity but also participates in the transmission of glutamate signalling through GluN/NMDA receptors^40^. The upregulation of ROR2 could therefore be part of the glutamatergic stress present in ALS. CBE treatment prevented the upregulation of ROR2 in SOD1^G86R^ mice.

DEPDC7 is a positive modulator of the NF-κB transcription factor and interacts with the CARMA proteins to the CBM Complex^41^. Due to the wide range of physiological processes regulated by NF-κB, a dysregulation of DEPDC7 could have repercussions on the activation of autophagy, inflammation, neuronal plasticity and cell survival^42, 43^. The strong upregulation of DEPDC7 by CBE treatment was confirmed by qPCR.

### CBE stimulates maturation of in vitro motor units and promotes recovery after sciatic nerve injury *in vivo*

**Figure 5:**
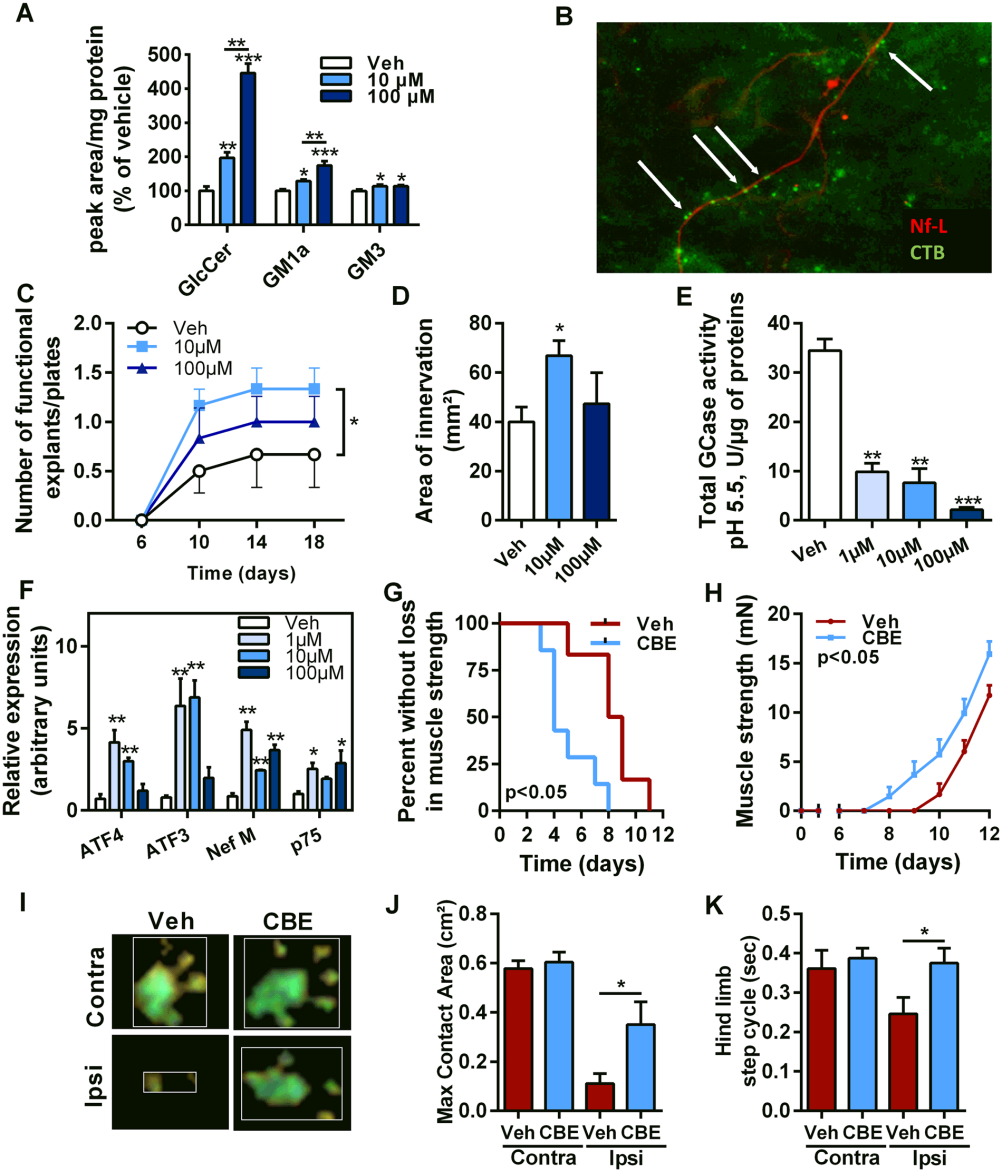
Inhibition of GlcCer degradation by CBE improves *in vitro* axonal plasticity and *in vivo* nerve regeneration. (**A**) Quantification of glycosphingolipids in the co-culture of muscle and spinal cord explants after CBE treatment. (**B**) Representative pictures for the distribution of neurofilament-L (red) and the cholera toxin subunit **B** (CTB, green) in the co-culture. Arrows indicate CTB-positive structures along an axon. (**C**) Given is the number of functional explants per culture plate identified by the presence of contraction of muscle fibres (n = 6). (**D**) Total area of innervation of functional explants after differentiation (n = 8/group). (**E**) Total GCase activity in PC12 cells after treatment with various doses of CBE (n = 3/group). (**F**) Transcriptional regulation of markers for neuronal differentiation in PC12 (n = 3/group). (**G**) Kaplan-Meier showing time to observable toe spreading after sciatic nerve injury (n = 7/group). (**H**) Muscle strength of ipsilateral hind paws, after sciatic nerve injury (n = 7/group). (**I**) Representative pictures for the areas of hind limb paws in contact with the ground. (**J**,**K**) Maximal contact area (**J**) and step cycle (**K**) of hind paws as determined by automated gait analysis. Mean +/− SEM; *p < 0.05; **p < 0.01.

Inhibition of GlcCer synthesis delays axonal regeneration after peripheral nerve injury^13^, suggesting that GlcCer is involved in the regenerative process of motor axons and in the maturation of NMJs. We hypothesized that increasing levels of GlcCer using CBE would have beneficial effects on the maturation of NMJs. CBE was added to a co-culture of spinal cord explants and myoblast cells, used as an *in vitro* model of motor units^44^. Levels of glycosphingolipids were upregulated upon CBE-treatment, in a dose-dependent manner for GlcCer and for the main neuronal ganglioside, GM1a (Fig. 5A). A modest effect was observed on the predominant muscle ganglioside GM3. As expected, CTB staining was localized predominantly with axons in the co-culture (Fig. 5B). Three weeks after differentiation, we observed a higher incidence of functional maturation of spinal explants (Fig. 5C) and greater total area of innervation per functional spinal explant (Fig. 5D) with a dose of 10 µM of CBE. A higher dose of CBE (100 µM) had no significant effect on these parameters compared to the control group receiving the vehicle. The effect of CBE on the regulation of gene expression during neuronal differentiation was studied in the neuronal PC12 cell line. CBE treatment led to a strong inhibition of GCase activity, almost complete at 100 µM of CBE (94% of inhibition), or moderate at lower doses of CBE, 1 µM and 10 µM, as shown by the presence of residual activity (Fig. 5E). The expression of the transcription factor atf3 and atf4 and the axonal proteins neurofilament-M and p75 were significantly upregulated by CBE at 1 μM and 10 μM. High dose of CBE had no significant effect on the expression of atf3 and atf4 **(**Fig. 5F **)**, suggesting that residual GCase activity is required for the mediation of axonal plasticity. Taken together, these results suggest a direct role for glycosphingolipids in the control of axonal plasticity and regeneration and could explain the positive effect seen on muscle innervation in SOD1^G86R^ mice after CBE treatment.

Next, we sought to determine whether CBE treatment could support motor recovery after nerve injury in wild type mice. CBE was administered on a daily basis (10 mg/kg/day), from 2 days prior injury until the end of the study, 12 days after injury. Recovery of motor function was assessed by monitoring the time to toe spreading, interpreted as the first visible signs of re-innervation, and by general locomotion and motor strength. In the vehicle group, toe spreading was observed 8 days after nerve injury, and recovery of muscle strength started 10 days after injury. At the study endpoint, 12 days after the injury, the locomotor profile of the vehicle group showed a strong reduction in the contact area and reduced hind limb step cycle of ipsilateral paw. Toe spreading and recovery of muscle strength occurred significantly faster by 2–4 days after CBE treatment (Fig. 5G,H), and the locomotor profile of the injured paw was closer to the contralateral side for contact area (Fig. 5I,J) and hindlimb step cycle compared to the vehicle group (Fig. 5K). Experimental denervation, induced by peripheral nerve injury, led to the severe loss of CTB-positive structures binding at the presynaptic part of the NMJs on the ipsilateral side of injury in tibialis anterior muscle, presumably due to the Wallerian degeneration or to lack of mature myelin sheaths. A partial loss of CTB staining was also observed at the post-synaptic part of the NMJ after denervation (Supp. Fig. 3 **)**. In all, partial inhibition of the GCase activity and increase in glycosphingolipids were beneficial for the regeneration of motor axons post-injury.

## Discussion

### Complex lipids participate in stability of neuromuscular junctions

Case reports from the 1980’s reported the presence of antibodies targeting gangliosides, and particularly GM1a, in the serum of some ALS patients^45, 46^ along with the presence of unusual gangliosides in post-mortem brains and spinal cords^47, 48^, emphasizing a role for glycosphingolipids in ALS. Administration of gangliosides was clinically tested in ALS patients with preliminary either positive or inconclusive effects, especially since these studies were underpowered^49–51^. Lipid microdomains are membrane areas enriched in cholesterol, sphingomyelin and glycosphingolipids (e.g. GM1a). These domains participate in cell-cell interaction^52^ and are key for the trafficking and clustering of acetylcholine receptors at the NMJs^53–55^. Depletion of cholesterol and sphingolipids from lipid rafts prevents the formation of acetylcholine receptors upon agrin application^56, 57^.

Acute motor axonal neuropathy, a subtype of Guillain-Barré disease, is due to the presence of autoimmune antibodies targeting GM1a at the motor endplate or at the nodes of Ranvier, causing specific dismantling of motor units with no influence on sensory functions. These autoimmune antibodies may prevent the interaction between the sugar headgroup of gangliosides with surrounding molecules. Indeed, the ganglioside GM1a interacts with neurotrophin receptors^58^, such as TrkA and modulates ions flux across membranes through the activation of Na+ -K+- ATPase exchanger^19^.

In the present study, we used the cholera toxin subunit B (CTB), which binds to gangliosides with highest affinity for GM1a^29, 30^. We show a loss of CTB staining in the immediate vicinity of motor axons and of muscle acetylcholine receptors, in early symptomatic SOD1^G86R^ mice, reinforcing the possibility that loss of gangliosides like GM1a may be a critical factor for muscle denervation in ALS.

### Clinical relevance of GCase inhibition for ALS

Modulators of sphingolipid levels have not been clinically tested in ALS and a therapy based on GCase activity appears attractive. We and others have reported increased gangliosides in ALS patients and SOD1 mice^13, 14^, and deleterious effects after administration of inhibitors of GlcCer synthesis for the integrity of neuromuscular junctions in SOD1^G86R^ mice^13^ and on their survival^14^. In addition, these studies have demonstrated that inhibition of GlcCer synthesis is also highly detrimental for axonal regeneration after peripheral nerve injury^13^. We propose that increases in glycosphingolipid levels seen in ALS patients and SOD1 mice are part of an innate and physiological, but late, response to neurodegeneration.

We show here that glycosphingolipids are intimately involved in innervation in normal mice, and reduced at neuromuscular junctions in the early stages of disease progression of SOD1^G86R^ mice.

We also report for the first time a beneficial effect of an inhibitor of GCase activity in an animal model of ALS. Indeed, partial inhibition of GCase activity in SOD1^G86R^ mice, with a modest dose of CBE, resulted in preserved motor functions, higher counts of motor neurons and preserved NMJs. In addition, the treatment was able to promote faster axonal elongation *in vitro* and *in vivo*, confirming that partial inhibition of GCase activity, associated with an increase in other glycosphingolipids, is beneficial for motor units.

Our study was designed using a low dose of CBE, which is a potent inhibitor of the lysosomal GCase with moderate but significant inhibition of the non-lysosomal GCase^35^. Gaucher disease is caused by homozygous mutations in GBA1, which are an important risk factor for Parkinson disease^59^, and mutations in the non-lysosomal GBA2 have been linked to hereditary spastic paraplegia^60^ and spastic ataxia^61^.The covalent binding of CBE to GCases may easily lead to full inhibition of total GCase activity if given in high enough dose, as shown in our *in vitro* assay. CBE has been used to induce a chemical model of Gaucher disease in neonatal mice^26^. However, the toxicity of CBE is very widely variable depending on mouse strain^62^. Even though lysosomal GCase is inhibited by 90–95% at a daily dose of 25 mg/kg/day starting at neonatal age, there is no correlation between lifespan and residual enzyme activity. A/J mice die within 25 days with severe motor defects, whereas BTBR + Ttftf mice live for more than 200 days. FVB mice, the background strain in our SOD1^G86R^ mice, live for about 120 days. A washout of 3 days following CBE treatment has been reported to be long enough to restore GCase activity^26^, showing the importance of partial inhibition. Optimal dosage for long-term administration of GCase inhibitors should be therefore carefully determined.

Gaucher disease patients have elevated plasma levels of GlcCer and gangliosiodes^63, 64^, suggesting that the GlcCer accumulated in the lysosome can get back to the early Golgi and be converted into lactosylceramide which can then be sialylated to GM3, a precursor of GM1a. As we saw an elevation of GM1a in the SOD1 mouse spinal cord after CBE treatment, it is possible that it is generated from GlcCer via the biosynthetic pathway in the Golgi.

The increase of GM1a could account for the beneficial effects of GCase inhibition in SOD1^G86R^ mice. Changes in gangliosides have also been described in other neurodegenerative diseases. Brains of Alzheimer’s disease patients have low levels of gangliosides^65, 66^ and GM1a distribution is abnormal, particularly in the human dentate gyrus^67^. Loss of GM1a was also documented close to nigral dopaminergic neurons in Parkinson’s disease patients^68^ and associated with reduced neurotrophic signalling^69^. Conversely, direct injection of GM1a in Parkinson’s disease patients stabilized disease progression in a pilot trial^70^. In a randomized and double-blind trial, direct administration of GM1a reduced motor symptoms while treatment discontinuation worsened symptoms emphasizing a direct effect^71^. Synthesis of GM1a is increased in cultured cortical neurons after glutamate-induced stress and reduce excitotoxicity^72^ and intracerebroventricular infusion of GM3 improved survival in SOD1 G93A mice^14^. Interestingly, a recent study reports the beneficial effect of IgM against GM1a in SOD1 mouse models. The authors proposed that the IgM treatment triggers neuroprotective cell signalling after the membrane reorganization of GM1a^73^.

Degeneration of NMJs precedes motor neuron loss in ALS^74^ and axonal transport is deficient in SOD1(G93A) mice before disease onset^75^. In our study, CTB staining was markedly reduced at innervated neuromuscular junctions of early symptomatic SOD1^G86R^ mice. Loss of gangliosides at distal motor axons may be an early pathological event in ALS. CTB staining was fully restored after CBE treatment, indicating that this is an effect on complex gangliosides and on innervation. Despite a 50% reduction of total GCase activity after low dose CBE treatment, we saw a 10% increase in gangliosides in the spinal cord, in line with previous reports^28, 76^. Neuroprotection detected after GCase inhibition could therefore be independent of spinal gangliosides. Differential expression of genes was previously reported in the transcriptome of isolated motor neurons^77^ and whole spinal cord^78–80^ of SOD1^G86R^ mice. Our RNA sequencing analysis revealed beneficial effect of CBE on the global spinal transcriptome of SOD1^G86R^ mice. Almost two thirds of the transcriptional dysregulations present at early symptomatic stage were not found after treatment. Notably, GCase inhibition by CBE prevented the deregulation of genes involved in molecular pathways known to participate to disease progression, including cell signalling, energy metabolism, neuroinflammation and axonal guidance. Importantly, our data support an effect of CBE on axonal plasticity in SOD1^G86R^ mice, with higher rate of innervated NMJs. We demonstrated an effect of CBE, most likely direct, on axonal regeneration *in vivo* on wild type mice, and *in vitro* on the expression profile of key transcription factors for axonal elongation and on building functional neuromuscular junctions. These experiments do not allow a definition of causalities but encourage further work on potential benefit of this approach.

To conclude, we now have evidence whereby inhibition of GCase preserves innervation and slows down disease progression in SOD1^G86R^ mice, whereas inhibition of GlcCer synthesis has exactly the reverse effects^13, 14^. In all, partial inhibition of GCase activity leading to increased levels of GlcCer and downstream glycosphingolipids is beneficial for the regeneration of motor axons post-injury in wild type mice and delays the degeneration of motor neurons and neuromuscular junctions in an animal model of ALS.

## Method

### Patients

A case–control study was performed to study the distribution of glycosphingolipids in the cerebrospinal fluid samples of 14 ALS patients and 12 control subjects. Individuals were recruited at the ALS centre in Tours, France. Cerebrospinal fluid was collected during routine lumbar puncture at the time of diagnosis from individuals with ALS, or from individuals showing motor dysfunctions. Controls were diagnosed with Parkinson’s disease (n = 1), cerebellar ataxia (n = 1), dementia (n = 1), spinal stenosis (n = 1), epilepsy (n = 1), axonal neuropathy (n = 4), and motor dysfunctions of unclear origin but unrelated to ALS (n = 3). CSF samples from age- and gender-matched healthy controls were inaccessible at the time of the study. All patients gave informed consent and the protocol was approved by the ethical committee of Tours. All human-related methods were performed in accordance with the relevant guidelines and regulations. Standard biochemical tests were performed, including bacteriology test, and glucose and protein quantifications.

### Animal experiments

Experiments followed current European Union regulations (Directive 2010/63/EU) and were performed by authorized investigators (A67–402 to A.H.), after approval by the ethic committee of the University of Strasbourg and by the ministry of higher education and research (APAFIS#662, APAFIS#2255).

#### SOD1^G86R^ mice

FVB/N female mice, overexpressing the SOD1(G86R) protein, were maintained in our animal facility at 23 °C with a 12 hours light/dark cycle. Mice had access to water and to regular A04 rodent chow ad libitum. GlcCer degradation was inhibited with conduritol B epoxide (CBE, 10 mg/kg/day, Cayman chemical, Ann Arbor, USA). CBE (10 mg/kg/d) or the vehicle (NaCl 4%) was given by intraperitoneal injection. The treatment started at 75 days of age and stopped at 95 days of age. Wild type littermates served as controls. Mice were sacrificed by intraperitoneal injection with ketamine chlorohydrate (100 mg/kg) and xylazine (5 mg/kg) and intracardially perfused with PBS at 4 °C.

#### Sciatic Nerve Injury

Peripheral nerve injury was performed in order to induce muscle denervation and axonal regeneration. Wild type mice were anesthetized with ketamine chlorohydrate (100 mg/kg) and xylazine (5 mg/kg). The sciatic nerve was exposed at mid-thigh level and lesioned with fine forceps for 30 s. The skin incision was sutured, and mice were allowed to recover. The hind limb, contralateral to the lesion, served as control. Mice were treated with CBE (10 mg/kg/d) for 12 days, starting the day before surgery. Mice were followed on a daily basis. Mice were sacrificed by intraperitoneal injection with ketamine chlorohydrate (100 mg/kg) and xylazine (5 mg/kg) and intracardially perfused with PBS at 4 °C.

#### Assessment of motor functions

Body mass and muscle strength (mean of three tests, grip test, Bioseb, Chaville, France) were analysed on a daily basis. Onset of muscle strength loss was defined as a drop of more than 10% of the mouse maximal strength.

The locomotor profiles of mice were analysed with a catwalk device (Noldus). Mice were habituated to the device at 85 days of age for SOD1^G86R^ mice, or before surgery for mice subjected to sciatic nerve crush. The locomotor profiles were recorded at least three times per mice. The software CatWalk XT (version 10.5, Noldus) was use to analyse video recording and to determine the average speed (average speed of an animal’s body during the whole run), paw swing (duration in seconds of the step corresponding to the absence of contact between a paw and the glass plate), stride length (distance between successive placements of the same paw), limb step cycle (duration between two consecutive initial contacts of the same paw), max contact area (surface area of the print at Max contact) and stand index were measured.

Electromyography: After anaesthesia as indicated above, muscle fibre potentials were detected using a electromyography apparatus (Dantec, Les Ulis, France), in accordance with the recommendation of the American Association of Electrodiagnostic Medicine. A concentric electromyography-needle electrode (no. 9013S0011, diameter 0.3 mm; Medtronic, Minneapolis, MN) was inserted in the gastrocnemius. Ground signal was determined with a monopolar needle electrode (no. 9013R0312, diameter 0.3 mm; Medtronic) inserted into the tail of the animal. Presence of fibrillations was investigated in three different muscle regions. Only spontaneous activity with peak-to-peak amplitude of at least 50 μV was considered to be significant.

### Cell culture

#### PC12

PC12 cells (Sigma-Aldrich) were cultured in 24-well plates with Dubelcco modified Eagle medium, 10% horse serum, 5% foetal calf serum, 1% penicillin/streptamicyine, and 1% Fungizone. At 70% of confluence, differentiation was initiated in DMEM, 1% horse serum, 1% foetal bovin serum, 1% penicillin/streptamicyine, 1% fungizone and nerve growth factor (100 ng/mL). CBE treatments started the first day of differentiation. Mediums were renewed every other day. Twelve days after differentiation, the cells were collected, pelleted by centrifugation and stored at −80 °C until further analysis.

#### Co-culture of spinal cord explants and myoblasts

The rat spinal cord-human muscle co-culture was performed as described previously^81^. Briefly, human myoblast was grown in a F14 medium (Invitrogen, Cergy-Pontoise, France), with glutamine 2 mM, insulin, epidermal growth factor, basic Fibroblast growth factor, foetal bovine serum 10% (FBS, Hyclone) and an antibiotic-antimycotic mixture (Gibco). Spinal cord explants of 14-day-old Wistar rat embryos (Janvier, Le Genest-St-IsIe, France) were placed on the muscle monolayer. The co-cultures were maintained in MEM (Gibco) supplemented with 25% medium 199 (Gibco), 5% FBD, 1 pg/ml insulin and antibiotic-antimycotic mixture. CBE treatment was initiated when the spinal cord explant were added to the muscle monolayer and was renewed twice a week for 3 weeks, until the end of the experiments. Innervation areas were identified by the presence of contraction of muscle fibres, highlighted under microscopy and analysed with ImageJ^82^.

### Histology

For histological analysis, tissues were fixed 1 hour in paraformaldehyde 4% and stored in PBS at 4 °C or snap frozen in isopentane and stored at −80 °C until further use.

#### Spinal cord

Lumbar segments L2-L3 fixed in paraformaldehyde 4% were used for studying the number of motor neurons innervating tibialis anterior muscle^83^. Coronal sections 40 µm thick from L2-L3 spinal segment were realized with a vibratome and were stained with an anti-choline acetylcholine transferase (1/100, Millipore, France) and an alexa594 conjugated goat (1/200, Jackson) antibodies. All neurons located in the ventral horn, that were >400 µm^2^ in size and ChAT positive were considered as alpha motor neurons. Six sections of spinal cord that were apart over a length of 0.24 mm were counted. Photomicrographs were taken with a Nikon microscope and cell area of motor neurons was measured with NIS Element 4.0 (Nikon).

#### Muscle bundles

Tibialis anterior muscle samples fixed in paraformaldehyde 4% were dissected into bundles under a binocular microscope. Bundles were collected from at least three different parts of the muscle. Acetylcholine receptors in the postsynaptic apparatus of NMJs were labelled with rhodamine-conjugated α-bungarotoxin (Sigma–Aldrich). Immunofluorescent labelling of nerve terminals was performed with a rabbit polyclonal anti-synaptophysin antibody (1/200, Abcam, Cambridge, UK) and with a rabbit polyclonal anti-neurofilament L antibody (1/200, Abcam, Cambridge, UK). For assessing NMJs integrity, an Alexa-488 conjugated goat anti-rabbit was used as secondary antibody (1/500, Jackson). Neuromuscular junction integrity was assessed by studying the colocalization of synaptophysin/neurofilament and α-bungarotoxin signal.

Effect of CBE on gangliosides at the neuromuscular junction was determined by labelling muscle bundles from tibialis anterior with cholera toxin sub-unit beta (CTB) coupled with an Alexa488 dye (1/200, ThermoFisher), and the rhodamine-conjugated α-bungarotoxin (1/500, Sigma-Aldrich). An Alexa-594 conjugated goat anti-rabbit was used as secondary antibody (1/500, Jackson). Only NMJs showing a normal axonal staining were considered for assessing CTB signal.

Wild type NMJs usually shows several dozens of CTB positive structures. CTB signal was considered as normal when at least 10 CTB positive structures were detected along presynaptic motor axons of innervated NMJs. Photomicrographs were taken with an ApoTom 2 (Zeiss) microscope and analysed with ZEN 2 (blue edition).

#### Coronal muscle sections

Twenty-micrometre sections were obtained by cutting isopentane fresh-frozen tibialis anterior muscle perpendicular to the muscle axis with a cryostat at −20 °C (Leica, Nanterre, France). Acetylcholine receptors in the postsynaptic apparatus of neuromuscular junctions were labelled with rhodamine-conjugated α-bungarotoxin (Sigma–Aldrich). Gangliosides distribution was labelled with the cholera toxin sub-unit beta coupled with an Alexa488 dye (1/200, ThermoFisher). Photomicrographs were taken with a Nikon microscope and analysed with NIS Element 4.0 (Nikon).

### Normal phase high performance liquid chromatography

Glycosphingolipids were studied in the cerebrospinal fluid of ALS patients, in mouse tissues and cells after CBE treatment. GlcCer and downstream gangliosides were analysed as described previously^13^. Tissues were homogenized in water using an Ultraturax T25 probe homogenizer (IKA, Germany). Lipids from tissue homogenates were extracted with chloroform and methanol. The glycosphingolipids were then further purified using solid-phase C18 columns (Telos, Kinesis, UK). After elution, the glycosphingolipids fractions were split in half, dried down under nitrogen at 42 °C and treated with either Cerezyme® (Genzyme, Cambridge, MA) to obtain glucose from GlcCer or ceramide glycanase (prepared in house from the medicinal leech Hirudo medicinalis/verbena) to obtain oligosaccharides from other glycosphingolipids. The liberated glucose and free glycans were then fluorescently-labelled with anthranillic acid (2-AA). To remove excess free label, samples were separated in DPA-6S SPE columns (Supelco, PA, USA). Purified 2AA-labeled glucose and 2AA-labeled oligosaccharides were separated and quantified by normal-phase high-performance liquid chromatography as previously described by Neville and colleagues^84^. The solid phase used was a 4.6 × 250 mm TSK gel-Amide 80 column (Anachem, Luton, UK). The HPLC system consisted of a Waters Alliance 2695 separations module and an in-line Waters 2475 multi λ-fluorescence detector set at 360 nm (excitation) and 425 nm (emission).

### β-glucosidase activity

Total β-glucosidase activity was assayed using the β-Glucosidase Activity Assay Kit (MAK129, Sigma). Dried tissues were lysed with a TissuLyser (Qiagen, CA) and suspended in ice-cold PBS. After centrifugation (14000 g for 10 min at 4 °C), the supernatants were transferred to new tubes and stored at −80 °C. Enzymatic reactions were carried out in KPi buffer (60 mM, pH 5.5) with p-NPG for 20 minutes at 37 °C. Final absorbance was measured at 405 nm.

### Analysis of spinal transcriptome

#### RNA sequencing

Frozen samples of spinal cord were homogenised using a TissuLyser (Qiagen, CA). Extraction of total RNA was performed using the RNeasy Plus Universal Tissue kit (Qiagen) accordingly to manufacturer recommendations. RNA quality was assessed with BioAnalyser (Agilent). Libraries of template molecules suitable for high throughput DNA sequencing were created using the TruSeq™ RNA Sample Preparation v2 Kit. Briefly, mRNA were purified from 600 ng of total RNA using poly-T oligo-attached magnetic beads and fragmented using divalent cations at 94 °C for 8 min. The cleaved mRNA fragments were reverse transcribed to cDNA using random primers then the second strand of the cDNA was synthesized using DNA Polymerase I and RNase H. The double stranded cDNA fragments were blunted using T4 DNA polymerase, Klenow DNA polymerase and T4 PNK. A single ‘A’ nucleotide was added to the 3′ ends of the blunt DNA fragments using a Klenow fragment (3′ to 5′ exo minus) enzyme. The cDNA fragments were ligated to double stranded adapters using T4 DNA Ligase. The ligated products were enriched by PCR amplification (30 s at 98 °C; [10 s at 98 °C, 30 s at 60 °C, 30 s at 72 °C] × 12 cycles; 5 min at 72 °C). Then surplus PCR primers were removed by purification using AMPure XP beads (Agencourt Biosciences, Fullerton, CA, USA). The libraries were loaded in a flow cell and clusters were generated in the Cbot and sequenced in an Illumina Hiseq 2500 sequencer as single-end 50 base reads. Image analysis and base calling were performed using RTA 1.18.61 and CASAVA 1.8.2. Reads were mapped onto mm10 assembly of mouse genome using Tophat v2.0.14^85^. Quantification of gene expression was performed using HTSeq v0.6.1^86^ and Ensembl release 81 database. After normalization, statistical analysis for differential gene expression has been performed using BRB-arrayTools v4.4.1, developed by Dr. Richard Simon and the BRB-ArrayTools Development Team. Difference among groups was assessed with ANOVA for fixed model and considered significant with a FDR set at 0.1, and after post-hoc analysis with the Tukey’s Honest Significant differences method and p value below 0.05. Heat map clustering was performed using JMP 11.0.0, with the Ward method applied to the normalized read counts of genes whose expression was significantly modulated upon CBE treatment. The colour code represents the level of expression of the genes (red: high level; black: medium level; green: low level). Gene ontology was performed using ConsensusPathwayDB^87, 88^ and the Reactom database^89, 90^.

#### Quantitative PCR

Gene expression was assessed on a CFX96 using SYBR green Supermix reagent (BioRad) according to manufacturer’s instructions. Relative quantification of each gene was determined using the Biorad software and normalized to the normalization factor generated from the reference genes (Pol2, TBP and 18S).

## Statistics

Experiments were performed by experimenters blinded for genotype and treatment identities, and the diagnosis of the patients. Data was expressed as the mean ± SEM and were analysed with PRISM 6.0b (GraphPad, San Diego, CA). Normality was assessed with Kolmogorov-Smirnov test or D’Agostino and Pearson. Two-tailed Student’s t test was used to compare two groups, and ANOVA followed by two-tailed Fisher’s LSD test was applied to compare more than two groups. If required, correction for multiple comparisons was performed using the Holm-Sidak method. Times to events were analysed with Log-rank test. Differences with p-values < 0.05 were considered significant.

### Dataset availability statement

All data generated or analysed during this study are included in this published article (and its Supplementary Information Files).

## Electronic supplementary material

SUPPLEMENTARY INFO
