# Supplementary material for: Inhibition of β-Glucocerebrosidase Activity Preserves Motor Unit Integrity in a Mouse Model of Amyotrophic Lateral Sclerosis
Source: Sci Rep. 2017 Jul 12;7:5235. doi: 10.1038/s41598-017-05313-0 (PMC5507914; doi:10.1038/s41598-017-05313-0)
Supplement: Supplementary file 1 — SUPPLEMENTARY INFO [file 41598_2017_5313_MOESM1_ESM.pdf]

Supplementary information

INHIBITION OF *β*-GLUCOCEREBROSIDASE ACTIVITY PRESERVES MOTOR UNIT INTEGRITY IN A MOUSE MODEL OF AMYOTROPHIC LATERAL SCLEROSIS

Alexandre Henriques [1, 2, 3], Mylene Huebecker [4], Hélène Blasco[5, 6], Céline Keime [7], Christian R Andres[5, 6], Philippe Corcia[5,8], David A Priestman [4], Frances M Platt [4], Michael Spedding[3] and Jean-Philippe Loeffler[1, 2 *]

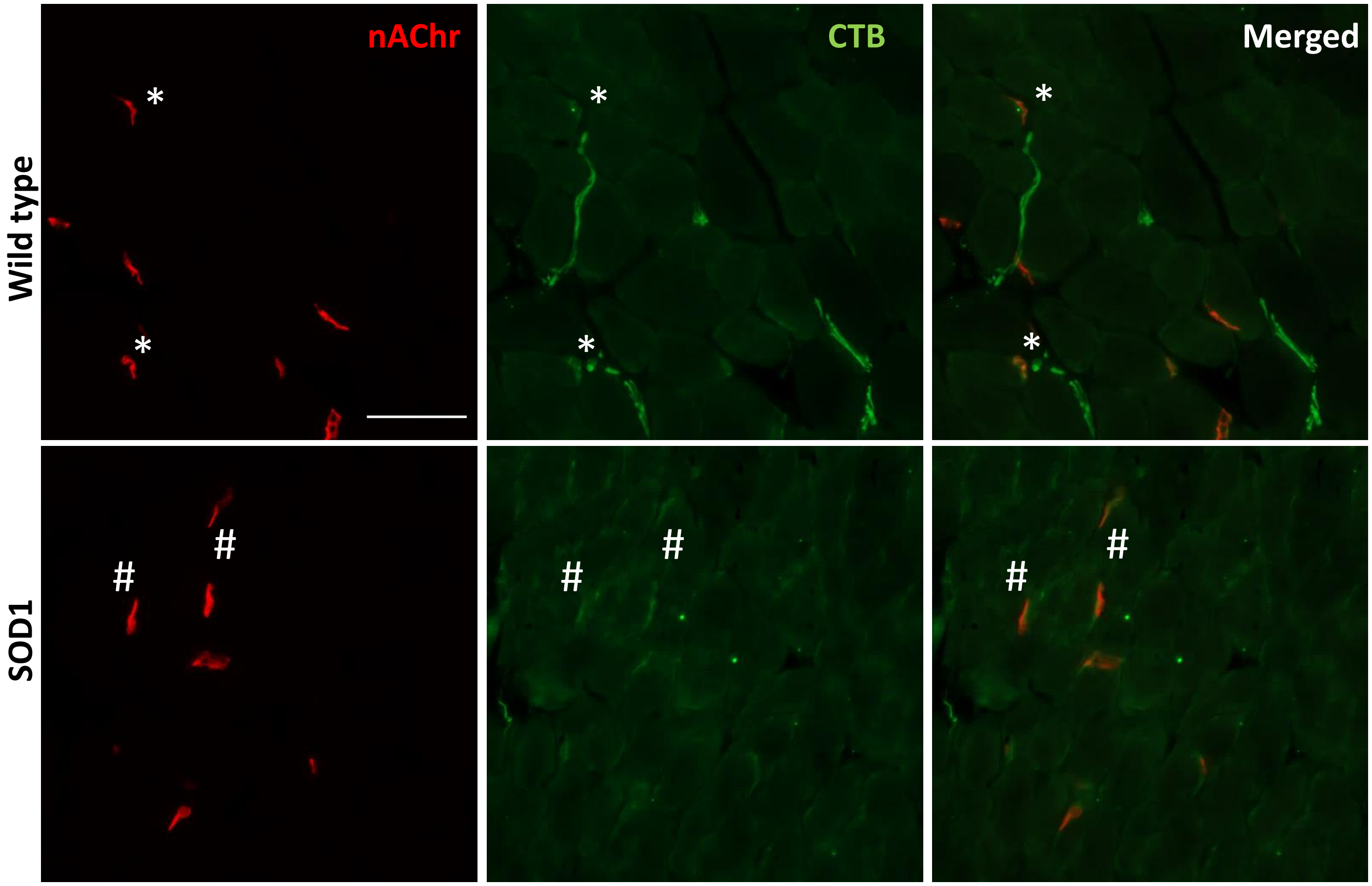

Supp Fig. 1. CTB signal on coronal muscle sections. CTB signal is observed in axons and at the synapse of the neuromuscular junctions in non-transgenic mice (*). Only a weak signal very close to the neuromuscular synapse is noticed in early symptomatic SOD1 mice (#). Scale bar 100µm

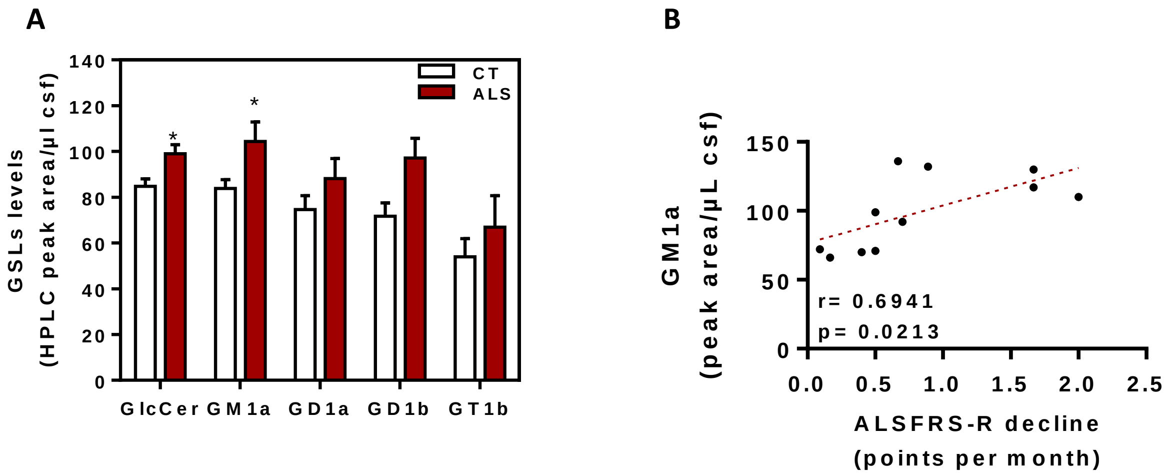

Supp Fig. 2. Glycosphingolipids in the cerebrospinal fluid.

A. Altered glycosphingolipid levels in the cerebrospinal fluid of ALS patients.

B. Correlation between GM1a level and disease progression in a subset of ALS patients.

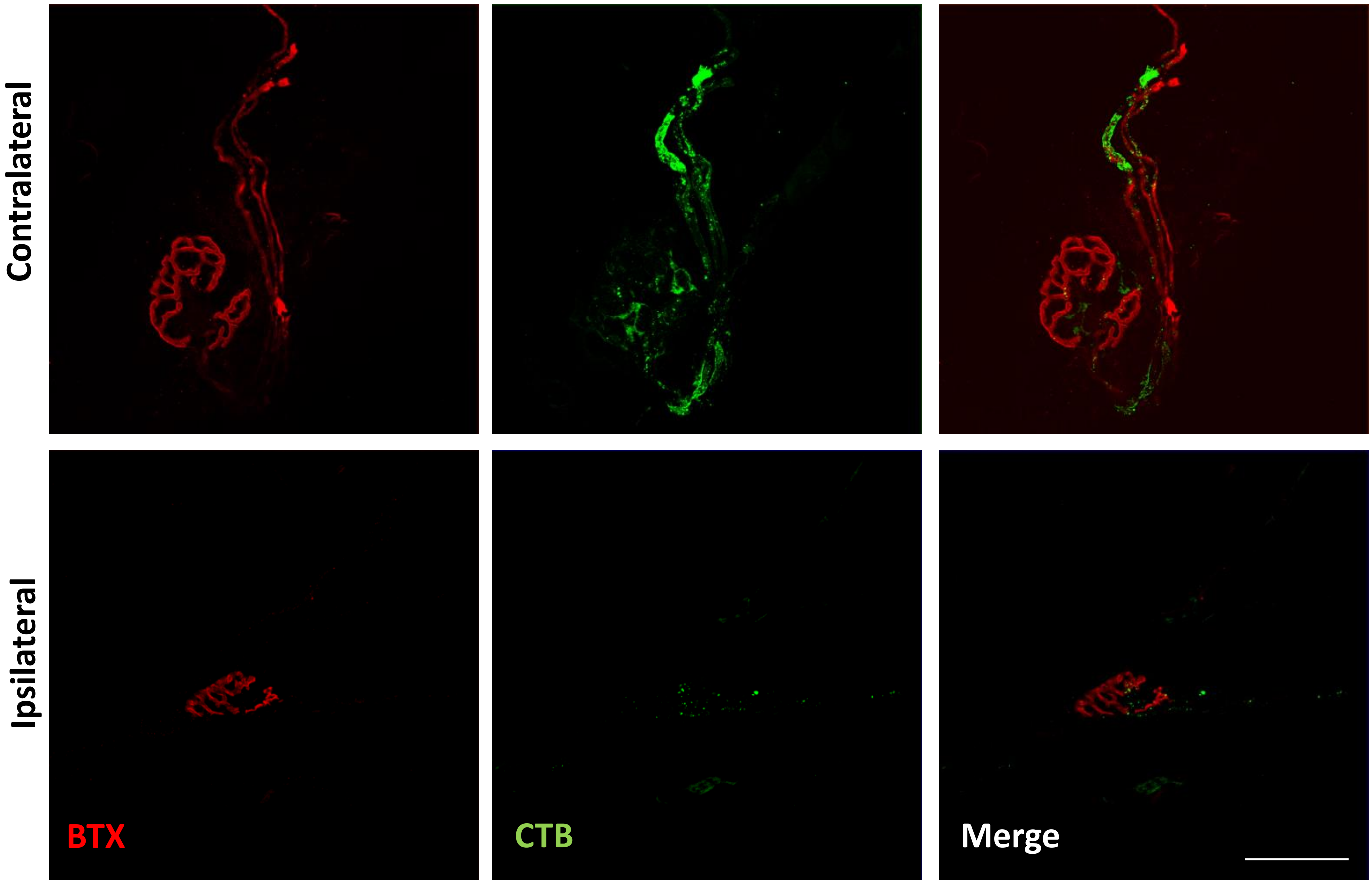

Supp Fig. 3. Loss of cholera toxin subunit B signal at the neuromuscular junction after sciatic nerve injury. Scale bar 50µm

pathway	p-value	q-value	source
Mitotic Anaphase	0.006	0.051	Reactome
Hemostasis	0.009	0.060	Reactome
RET signaling	0.020	0.076	Reactome
IGF1R signaling cascade	0.030	0.076	Reactome
Signaling by Insulin receptor	0.037	0.079	Reactome
Axon guidance	0.040	0.079	Reactome

Supp Table 1. Biological pathways modulated in the spinal cord of SOD*1*^{G86R} mice after CBE treatment
